# A comprehensive model to predict severe acute graft-versus-host disease in acute leukemia patients after haploidentical hematopoietic stem cell transplantation

**DOI:** 10.1186/s40164-022-00278-x

**Published:** 2022-05-03

**Authors:** Meng-Zhu Shen, Shen-Da Hong, Rui Lou, Rui-Ze Chen, Xiao-Hui Zhang, Lan-Ping Xu, Yu Wang, Chen-Hua Yan, Huan Chen, Yu-Hong Chen, Wei Han, Feng-Rong Wang, Jing-Zhi Wang, Kai-Yan Liu, Xiao-Jun Huang, Xiao-Dong Mo

**Affiliations:** 1 Peking University People’s Hospital, Peking University Institute of Hematology, National Clinical Research Center for Hematologic Disease, Beijing Key Laboratory of Hematopoietic Stem Cell Transplantation, No. 11 Xizhimen South Street, Xicheng District, Beijing, 100044 China; 2 National Institute of Health Data Science at Peking University, Peking University Health Science Center, Beijing, 100191 China; 3 Department of Hematology, Jiangsu Province Hospital, The First Affiliated Hospital of Nanjing Medical University, Jiangsu, 210036 China; 4 Peking-Tsinghua Center for Life Sciences, Academy for Advanced Interdisciplinary Studies, Peking University, Beijing, 100044 China; 5 Research Unit of Key Technique for Diagnosis and Treatments of Hematologic Malignancies, Chinese Academy of Medical Sciences, Beijing, 2019RU029 China

**Keywords:** Acute leukemia, Acute graft-versus-host disease, Haploidentical donor, Hematopoietic stem cell transplant, Predicted model

## Abstract

**Background:**

Acute graft-versus-host disease (aGVHD) remains the major cause of early mortality after haploidentical related donor (HID) hematopoietic stem cell transplantation (HSCT). We aimed to establish a comprehensive model which could predict severe aGVHD after HID HSCT.

**Methods:**

Consecutive 470 acute leukemia patients receiving HID HSCT according to the protocol registered at https://clinicaltrials.gov (NCT03756675) were enrolled, 70% of them (n = 335) were randomly selected as training cohort and the remains 30% (n = 135) were used as validation cohort.

**Results:**

The equation was as follows: Probability (grade III–IV aGVHD) = $\frac{1}{{1 + \exp \left( { - \,{\text{Y}}} \right)}}$, where Y = –0.0288 × (age) + 0.7965 × (gender) + 0.8371 × (CD3 + /CD14 + cells ratio in graft) + 0.5829 × (donor/recipient relation) − 0.0089 × (CD8 + cell counts in graft) − 2.9046. The threshold of probability was 0.057392 which helped separate patients into high- and low-risk groups. The 100-day cumulative incidence of grade III–IV aGVHD in the low- and high-risk groups was 4.1% (95% CI 1.9–6.3%) versus 12.8% (95% CI 7.4–18.2%) (*P* = 0.001), 3.2% (95% CI 1.2–5.1%) versus 10.6% (95% CI 4.7–16.5%) (*P* = 0.006), and 6.1% (95% CI 1.3–10.9%) versus 19.4% (95% CI 6.3–32.5%) (*P* = 0.017), respectively, in total, training, and validation cohort. The rates of grade III–IV skin and gut aGVHD in high-risk group were both significantly higher than those of low-risk group. This model could also predict grade II–IV and grade I–IV aGVHD.

**Conclusions:**

We established a model which could predict the development of severe aGVHD in HID HSCT recipients.

**Supplementary Information:**

The online version contains supplementary material available at 10.1186/s40164-022-00278-x.

## Introdution

Allogeneic hematopoietic stem cell transplantation (allo-HSCT) is the most important curative method for acute leukemia (AL), which can significantly improve the long-term survival [1, 2]. Human leukocyte antigen (HLA) haploidentical related donors (HIDs) have become one of the most important donors, which accounted for the proportion at 42% among allo-HSCT from family donors in Europe [3], and accounted for the proportion at 60% among all of the allo-HSCT in China [4].

Although many strategies [e.g., antithymocyte globulin (ATG) and post-transplant cyclophosphamide (PTCy)] are used to prevent acute graft-versus-host disease (aGVHD), it is still inevitable [5]. Only half of aGVHD patients could achieve durable responses to initial corticosteroid therapy [6], and there is no standard therapy for steroid refractory aGVHD and the survival among these patients is poor [7]. Thus, severe aGVHD remains the major cause of early mortality after HID HSCT [8–10]. An early-warning method for severe aGVHD can help to provide risk-stratification directed prophylaxis for aGVHD and significantly improve the survival of patients receiving HID HSCT.

Several demographic and transplant characteristics, such as patient age, underlying disease (e.g., chronic myeloid leukemia), comorbidities before allo-HSCT, donor/recipient gender mismatching (i.e., female donor/male recipient combination), donor and recipient cytomegalovirus (CMV) serostatus, donor type (i.e., HLA‐non‐identical donors), HLA disparity, and GVHD prophylaxis methods are reported as important risk factors for aGVHD [11, 12]. Particularly, donor/recipient relation [i.e., collateral relative donors (CRDs) [13] and maternal donors (MDs)] [14, 15] is associated with aGVHD after HID HSCT with ATG or PTCY for GVHD prophylaxis.

In addition, graft composition may be associated with aGVHD after allo-HSCT. For example, the CD4+/CD8+ T cells ratio in granulocyte colony-stimulating factor (G-CSF)-mobilized bone marrow (G-BM) [16] or the CD3+/CD14+ cells ratio in G-CSF-primed peripheral blood (G-PB) [17] can predict aGVHD after HID HSCT. However, most of the studies only reported the risk factors for aGVHD, and there was no comprehensive model which included the characteristics of demographic, disease, transplant, and graft composition for aGVHD prediction.

Thus, in the present study, we aimed to establish a comprehensive model which could predict the severe aGVHD in patients receiving HID HSCT with ATG for GVHD prophylaxis.

## Patients and methods

### Study design

Consecutive AL patients receiving HID HSCT between January 21, 2020 and May 31, 2021 at Peking University, Institute of Hematology (PUIH) were enrolled. The end point of the last follow-up for all survivors was November 11, 2021. A total of 67 patients had been previously reported by Ma et al. [18], and all of them were further followed-up. All patients were treated according to the protocol registered at https://clinicaltrials.gov (NCT03756675). Informed consent was obtained from all patients or their guardians. The study was conducted in accordance with the *Declaration of Helsinki*, and the protocol was approved by the Institutional Review Board of Peking University People’s Hospital.

### Transplant regimens

Major conditioning regimen consisted of cytarabine, busulfan, cyclophosphamide, and semustine [19, 20]. Twelve patients received total body irradiation (TBI)-based conditioning regimen. G-PB harvests were administered to the recipients on the same day of collection [18]. ATG, cyclosporine A, mycophenolate mofetil, and short-term methotrexate were administered to prevent GVHD. Particularly, patients with CRDs or MDs could receive low dose cyclophosphamide after transplantation based on ATG for GVHD prophylaxis (Additional file 1: Additional methods) [21].

### Evaluation of graft composition

The methods for graft composition evaluation were showed in Additional file 1: Additional methods [16, 22].

### Definitions

The definitions for disease risk index (DRI), engraftment, aGVHD, relapse, mortality, and survival were showed in Additional file 1: Additional methods [23–25].

### Building machine learning models

**Fig. 1:**
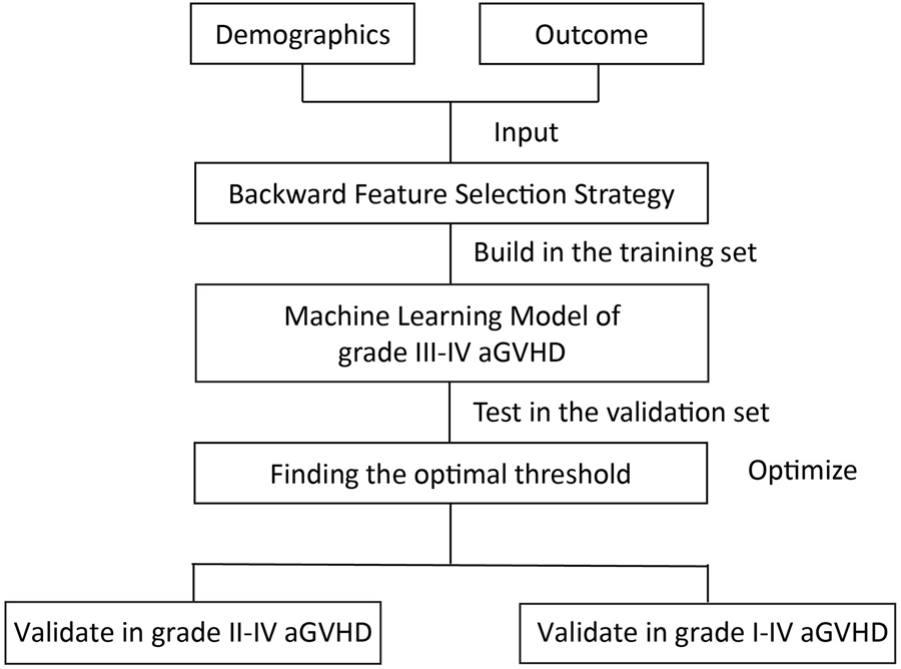
Flow diagram of building machine learning model

Our method consisted of three steps: selecting features, building models, and finding the optimal threshold (Fig. 1 and Additional file 1: Additional methods).

#### Backward feature selection strategy

We randomly selected 70% of the entire population (n = 335) as the training cohort, the remains 30% were used as validation cohort (n = 135). For primary outcome (i.e., grade III-IV aGVHD), the model building steps were performed in the training cohort and validated in the validation cohort. The sensitivity, specificity, area under curve score, and accuracy score were identified in both the training and validation cohort.

We used feature selection techniques to select the predictive variables (Additional file 1: Additional methods) [26]. By doing this, we could reduce the complexity of machine learning model, while also improve the generalizability. We set age and gender to be obligate variables in the machine learning model. For other variables, we selected top-3 significant variables using backward feature selection strategy. In detail, we started with all variables including age and gender. At each iteration, we removed the least significant variable (variable with the highest *P*-value) except age and gender. Aside from the involved variables, we also added an extra constant variate to make the feature selection more robust. The selection was realized using generalized linear models with binomial exponential family distribution of statsmodels v0.13.0 statistical models module with Python 3.8 based on anaconda3 development platform [27].

#### Building models

We used generalized linear models with binomial exponential family distribution to realize logistic regression models, which were equivalent models. Aside from the selected variables, we added an extra constant variate for the predicted model to make the machine learning models stronger. We used statsmodels v0.13.0 with Python 3.8 to build the models based on anaconda3 development platform. The model parameters were set to be the defaults [28–30].

#### Finding the optimal threshold

Logistic regression model produced values between 0 and 1, which could be treated as the probabilities to be positive prediction. We needed to determine the threshold of output positive predictions (1) or negative predictions (0). In detail, we drew Receiver Operating Characteristic (ROC) curves [31] and calculated the g-mean for each threshold [32]. The best threshold corresponded to the largest g-mean. The g-mean was calculated as sqrt [tpr × (1 − fpr)], where tpr represented true positive rate, fpr represented false positive rate, under a given threshold.

#### Evaluation for model

ROC-AUC was defined as the area under the curve of the true positive rate versus the false positive rate at various thresholds ranging from zero to one. Confusion matrix was a summary table of predictions. In this paper, the confusion matrix was of two-by-two shape. The diagonal showed the count values of correct predictions, while the others showed the count values of incorrect predictions. Besides, we also normalized the count values by the number of True Label (Outcome) or the number of Predicted Label (Prediction). To better visualize the matrix, we colored the values with Blues colorbar.

### Statistical methods

In the present study, the primary outcome was grade III to IV aGVHD. The secondary outcomes included grade II to IV aGVHD, grade I to IV aGVHD, relapse, non-relapse mortality (NRM), leukemia-free survival (LFS), and overall survival (OS).

Mann–Whitney *U*-test was used to compare continuous variables, χ^2^ and Fisher’s exact tests were used for categorical variables. The Kaplan–Meier method was used to estimate the probability of LFS and OS. Competing risk analyses were performed to calculate the cumulative incidence of aGVHD, relapse, and NRM [33]. Testing was two-sided at the *P* < 0.05 level. Statistical analysis was performed on SPSS 22.0 software (SPSS, Chicago, IL), and R software (version 4.0.0) (http://www.r-project.org).

## Results

### Patient characteristics

A total of 470 patients were enrolled, and the characteristics were all comparable between training and validation cohort (Table 1). All patients achieved neutrophil engraftment and the median time from HSCT to neutrophil engraftment was 12 days (range 9–28) days. Four hundred and fifty-eight (97.4%) patients achieved platelet engraftment and the median time from HSCT to platelet engraftment was 13 days (range 7–144) days, respectively.

**Table 1:** Patient characteristics

Characteristics	Training cohort (*n* = 335)	Validation cohort (*n* = 135)	*P* value
Median age at allo-HSCT, years (range)	28 (1–66)	31 (1–64)	0.596
Gender, *n* (%)			0.635
Male	198 (59.1)	83 (61.5)	
Female	137 (40.9)	52 (38.5)	
Underlying disease, *n* (%)			0.704
Acute myeloid leukemia	187 (55.8)	78 (57.8)	
Acute lymphoblastic leukemia	143 (42.77)	55 (40.7)	
Mixed-phenotype acute leukemia	5 (1.55)	2 (1.5)	
Disease status before allo-HSCT, *n* (%)			0.535
CR1	321 (95.8)	131 (97.0)	
> CR1	14 (4.2)	4 (3.0)	
Disease risk index before allo-HSCT, *n* (%)			0.714
Low and intermediate risk	268 (80.0)	110 (81.5)	
High and very high risk	67 (20.0)	25 (18.5)	
Donor/recipient relation, *n* (%)			0.379
Mother donor	26 (7.8)	12 (8.9)	
Collateral donor	12 (3.6)	0 (0.0)	
Others	297 (88.7)	123 (91.1)	
Donor/recipient gender matched, *n* (%)			0.258
Female donor/male recipient combination	57 (17.0)	29 (21.5)	
Others	278 (83.0)	106 (78.5)	
HCT-CI scores before allo-HSCT, *n* (%)			0.121
0 (Low-risk)	237 (70.7)	105 (77.8)	
1–2 (Intermediate-risk)	74 (22.1)	23 (17.0)	
≥ 3 (High-risk)	24 (7.2)	7 (5.2)	
Median donor age at allo-HSCT, years (range)	40 (9–70)	36 (10–63)	0.094
Cytomegalovirus serostatus before HSCT, *n* (%)			0.501
Donor +/recipient +	312 (93.1)	128 (94.8)	
Donor +/recipient −	11 (3.3)	3 (2.2)	
Donor −/recipient +	10 (3.0)	4 (3.0)	
Donor −/recipient −	2 (0.6)	0 (0.0)	
Number of HLA-A, HLA-B, HLA-DR mismatches, *n* (%)			0.914
1 Locu	8 (2.4)	3 (2.2)	
≥ 2 Loci	327 (97.6)	132 (97.8)	
Blood group compatibility, *n* (%)			0.719
Matched	175 (52.2)	73 (54.1)	
Mismatched	160 (47.8)	62 (45.9)	
Conditioning regimen, *n* (%)			0.350
Chemotherapy-based regimen	325 (97.0)	133 (98.5)	
TBI-based regimen	10 (3.0)	2 (1.5)	
Cell type, median count (range)			
MNC counts (× 10^8^/kg)	9.2 (4.4–27.3)	9.3 (4.2–27.5)	0.218
CD34+ cell counts (× 10^6^/kg)	3.8 (0.7–25.33)	3.9 (1.1–29.4)	0.572
CD3+ cell counts (× 10^6^ kg)	340.9 (116.2–874.2)	352.0 (170.4–1172.2)	0.617
CD4+ cell counts (× 10^6^/kg)	182.5 (68.3–600.1)	184.7 (75.2–492.7)	0.688
CD8+ cell counts (× 10^6^/kg)	126.7 (29.6–347.9)	128.0 (46.1–1511.2)	0.559
CD14+ cell counts (× 10^6^/kg)	211.3 (73.3–1065.0)	215.6 (95.8–716.9)	0.373
CD8+/CD3+ cells ratio	0.4 (0.2–0.7)	0.4 (0.1–1.3)	0.817
CD4+/CD8+ cells ratio	1.5 (0.4–4.7)	1.5 (0.3–3.0)	0.672
CD4+/CD3+ cells ratio	0.6 (0.2–0.8)	0.5 (0.1–0.7)	0.627
CD3+/CD14+ cells ratio	1.6 (0.6–4.4)	1.5 (0.6–3.7)	0.601
Median follow-up of survivors, days (range)	203 (62–490)	192 (52–509)	0.134

allo-HSCT, allogeneic hematopoietic stem cell transplantation; CR, complete remission; HLA, human leukocyte antigen; HCT-CI, hematopoietic cell transplantation-specific comorbidity index; MNC, mononuclear cells; TBI, total body irradiation

Two hundred and sixty-six (56.6%), 129 (27.4%), and 33 (7.0%) patients experienced grade I to IV aGVHD, grade II to IV aGVHD, and grade III to IV aGVHD after allo-HSCT, respectively. The median time from HSCT to aGVHD was 20 days (range 8–99) days. The cumulative incidence of grade I to IV aGVHD, grade II to IV aGVHD, and grade III to IV aGVHD at 100 days after HID HSCT was 56.5% (95% CI 52.0–61.0%), 27.3% (95% CI 23.3–31.3%), and 6.8% (95% CI 4.5–9.1%), respectively.

Thirty-eight (8.1%) patients experienced relapse, and 16 (3.4%) patients died of NRM. Four hundred and forty-nine patients survived until the last follow-up, and the median duration of follow-up was 200 days (range 52 to 509) days. The probabilities of relapse, NRM, LFS, and OS at 100 days after HID HSCT were 2.8% (95% CI 1.3–4.3%), 1.5% (95% CI 0.4–2.6%), 95.7% (95% CI 93.9–97.6%), and 97.8% (95% CI 96.5–99.2%), respectively.

### Predicted model for grade III to IV aGVHD (model 1)

A predictive model for grade III-IV aGVHD was developed (Additional file 1: Additional methods, Table S1 and Fig. S1), and the equation was as follows:

$${\text{Probability}}\left( {{\text{grade III}}{-}{\text{IV aGVHD}}} \right) = \frac{1}{{1{ } + {\text{ exp}}\left( { - {\text{Y}}} \right)}}$$

where, Y = − 0.0288 × (age) + 0.7965 × (gender) + 0.8371 × (CD3 + /CD14 + cells ratio in graft) + 0.5829 × (donor/recipient relation) − 0.0089 × (CD8 + cell counts in graft) − 2.9046. Particularly, donor/recipient relation included immediate relative donors (IRDs) other than MDs (value = 0), MDs (value = 1), and CRDs (value = 2). Gender included male (value = 0) and female (value = 1). The age (years), CD8 + cell counts (× 10^6^/kg), CD3+/CD14+ cells ratio in graft used actual numerical value (Additional file 1: Table S1). The threshold of probability was 0.057392 and the g-mean was 0.682. Patients were separated into low- and high-risk groups by the threshold.

**Fig. 2:**
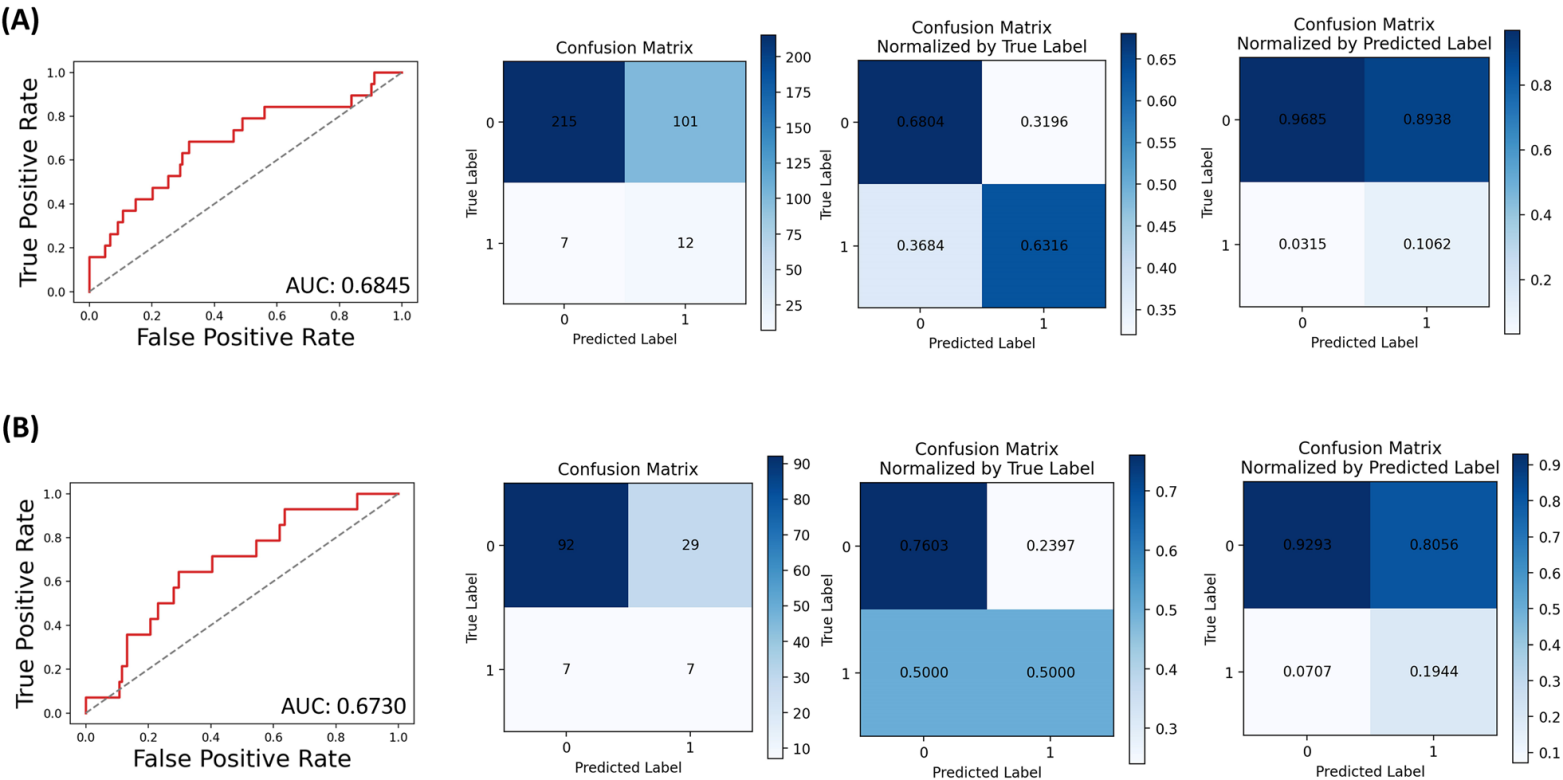
ROC curve and confusion matrix for grade III to IV aGVHD model in the training (**A**) and validation cohort (**B**)

In the training cohort, the sensitivity, specificity, area under curve score, and accuracy score were 0.632, 0.680, 0.685, and 0.678, respectively. ROC curve for the model and confusion matrix is shown in Fig. 2A and Additional file 1: Table S2. In the validation cohort, the sensitivity, specificity, area under curve score, and accuracy score were 0.500, 0.760, 0.673, and 0.733, respectively. ROC curve for the model and confusion matrix is shown in Fig. 2B and Additional file 1: Table S3.

### Verifying the predicted model in validation and total cohort

**Fig. 3:**
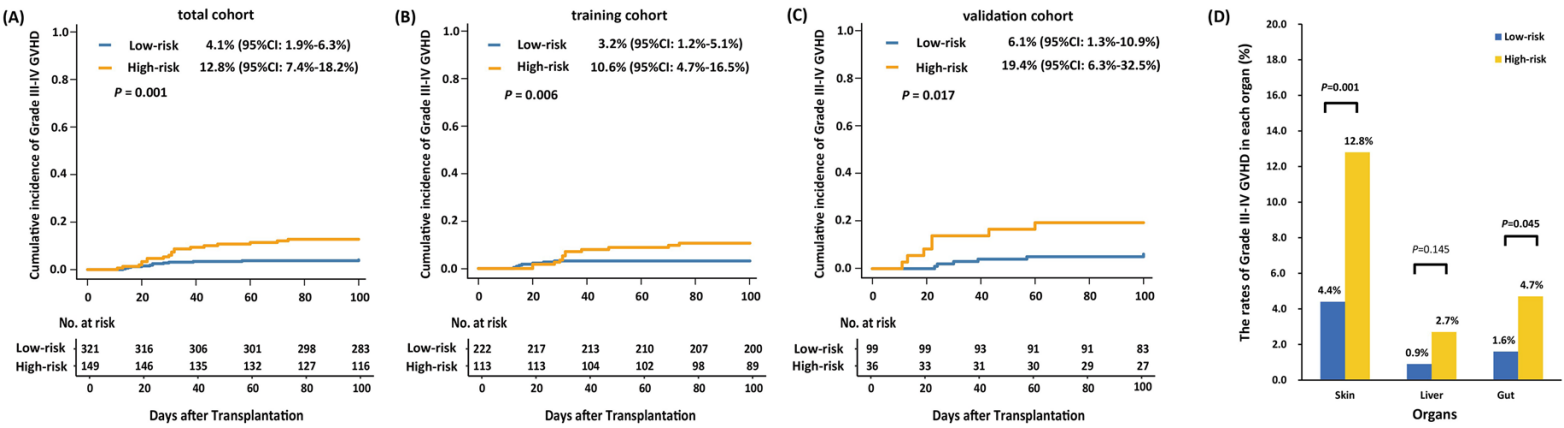
The 100-day cumulative incidence of grade III to IV aGVHD in the low- and high-risk groups in total (**A**), training (**B**), and validation (**C**) cohort, and **D** the rates of grade III to IV aGVHD of each organ in the low- and high-risk group

The 100-day cumulative incidence of grade III-IV aGVHD in the low- and high-risk groups was 4.1% (95% CI 1.9–6.3%) versus 12.8% (95% CI 7.4–18.2%) (*P* = 0.001), respectively, in total cohort (Fig. 3A).

The 100-day cumulative incidence of grade III-IV aGVHD in the low- and high-risk groups was 3.2% (95% CI 1.2–5.1%) versus 10.6% (95% CI 4.7–16.5%) with *P* = 0.006 and 6.1% (95% CI 1.3–10.9%) versus 19.4% (95% CI 6.3–32.5%) with *P* = 0.017, respectively, in training cohort (Fig. 3B) and validation cohort (Fig. 3C). The 100-day cumulative incidence of grade III-IV aGVHD in the low- and high-risk groups was 4.9% (95% CI 2.1–7.7%) versus 11.1% (95% CI 5.2–17.0%) with *P* = 0.033 and 2.1% (95% CI 0.0–4.9%) versus 18.8% (95% CI 5.0–32.5%) with *P* < 0.001, respectively, in patients with HCT-CI scores of 0 (Additional file 1: Fig. S2) and ≥ 1 (Additional file 1: Fig. S3).

The rates of grade III to IV skin and gut aGVHD in low-risk group were both significantly lower than those of high-risk group (skin: 4.4% *vs*. 12.8%, *P* = 0.001; gut: 1.6% *vs*. 4.7%, *P* = 0.045) (Fig. 3D).

### Validation of the predicted model in grade II to IV aGVHD

**Fig. 4:**
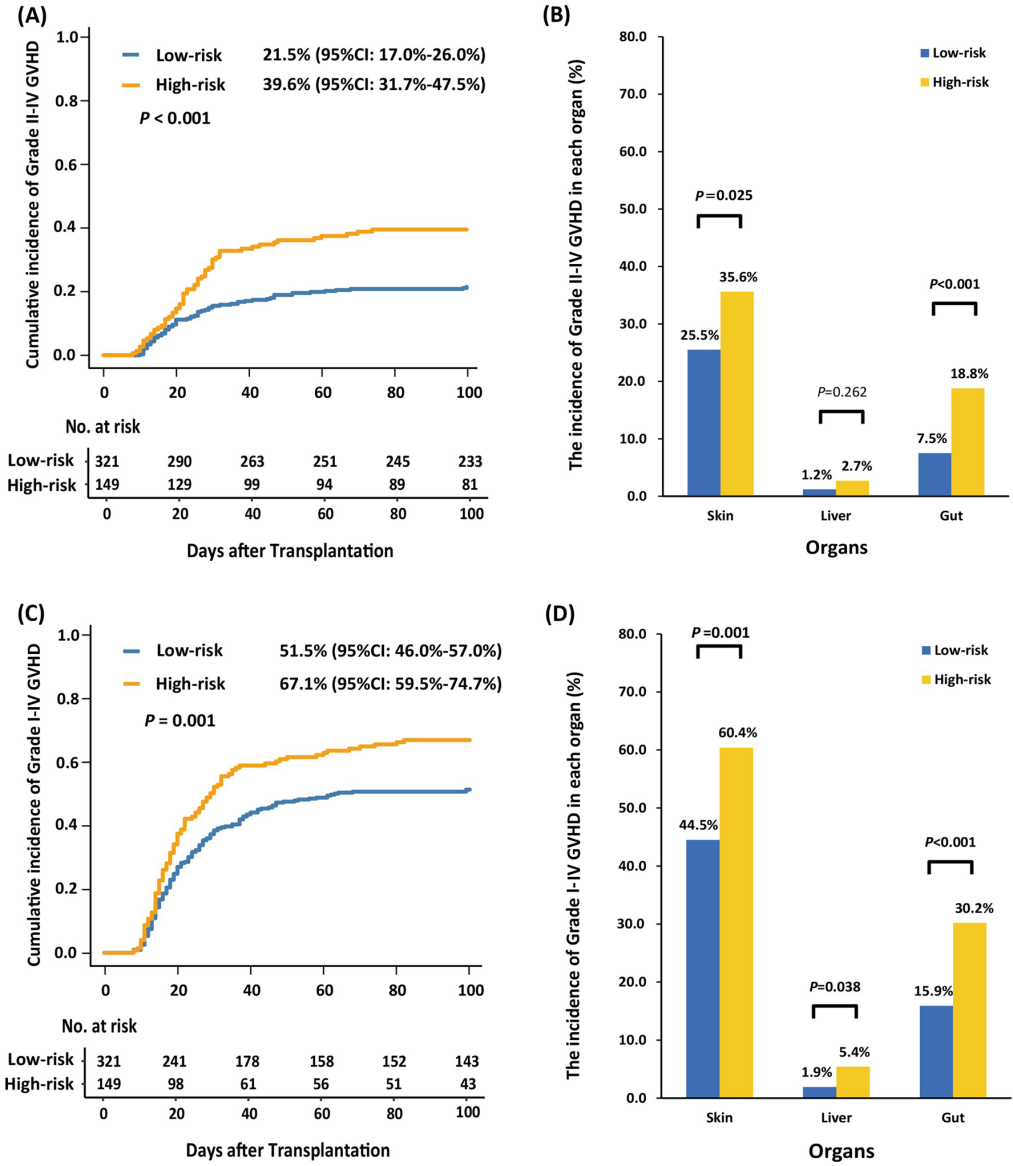
The association between predicted model and other GVHD endpoint in total population. **A** The 100-day cumulative incidence of grade II to IV aGVHD in the low- and high-risk groups; **B** The rate of grade II to IV aGVHD of each organ in the low- and high-risk groups; **C** The 100-day cumulative incidence of grade I–IV aGVHD in the low- and high-risk groups; **D** The rate of grade I to IV aGVHD of each organ in the low- and high-risk groups

In the total population, the 100-day cumulative incidence of grade II to IV aGVHD in the low-risk group and high-risk group was 21.5% (95% CI 17.0–26.0%) and 39.6% (95% CI 31.7–47.5%), respectively (*P* < 0.001, Fig. 4A). The rates of grade II to IV skin and gut aGVHD in the low-risk group were both significantly lower than those of high-risk group (skin: 25.5% *vs.* 35.6%, *P* = 0.025; gut: 7.5% *vs.* 18.8%, *P* < 0.001) (Fig. 4B).

### Validation of the predicted model in grade I to IV aGVHD

In total population, the 100-day cumulative incidence of grade I to IV aGVHD in the low-risk group and high-risk group was 51.5% (95% CI 46.0–57.0%) and 67.1% (95% CI 59.5–74.7%), respectively (*P* = 0.001, Fig. 4C). The rates of grade I to IV skin, gut, and liver aGVHD in the low-risk group were all significantly lower than those of high-risk group (skin: 44.5% *vs.* 60.4%, *P* = 0.001; gut: 15.9% *vs.* 30.2%, *P* < 0.001; liver: 1.9% *vs.* 5.4%, *P* = 0.038) (Fig. 4D).

### Validation of the predicted model in other clinical outcomes after HSCT

In total population, the probabilities of relapse, NRM, LFS, and OS at 100 days after HID HSCT were all comparable between the low- and high-risk groups in the total population (Additional file 1: Fig. S4).

## Discussion

In the present study, we established a predicted model for grade III to IV aGVHD including patient age, gender, donor/recipient relation, CD8+ T cell count, and CD3+/CD14+ cells ratio in the graft in training cohort, which was verified in validation and total cohorts. To the best of our knowledge, we firstly established a comprehensive model which can effectively predict severe aGVHD in HID HSCT recipients with ATG for GVHD prophylaxis.

Although some studies reported several risk factors of aGVHD, most of them did not integrate these factors and single factor may not provide comprehensive prediction for aGVHD. For example, Yahng et al. [34] reported that CD8+ cell counts in G-PB were associated with the occurrence of severe aGVHD after haplo-HSCT, which was not supported by the study of Liu et al. [17] In addition, MDs showed a higher risk of aGVHD compared with other IRDs in patients receiving ATG [14] or PTCY [15] for GVHD prophylaxis. In addition, we observed that the risk of aGVHD in CRDs group was as high as that of MDs group [13]. However, some authors reported that MDs did not increase the risk of aGVHD in patients using TCD protocol [35]. In the present study, the predictive model created by machine learning models is more accurate and reliable because it can eliminate the influence of selection bias in choosing variables. It also accounts for interaction and confounding factors, which cannot be completely adjusted for or eliminated using conventional statistics.

Compared with the traditional logistic regression model, the method proposed in this paper has several improvements. First, this method adds a feature selection step [26, 27]. We propose a backward feature selection strategy based on multi-factor analysis. This strategy is in a step-wise manner, which can ensure the stability of the feature selection process, and makes the model more generalizable. Second, in the model optimization process, we add a penalty function of the regularization term. It can reduce the risk of overfitting the training data, and further make the model more generalizable. Third, we consider the imbalance of positive and negative samples of the data when outputting the final prediction results. Hence, the traditional threshold of 0.5 is not directly used. Instead, we calculate the optimal threshold based on g-means index from the ROC curve [31, 32].

According to the theory of machine learning, adding more variables increases the capacity and performance upper bound of the predictive model [36, 37], but also increases the complexity of the predictive model. Additionally, many variables may make a model too difficult to clinically apply. Thus, obligate variables seem to be a balanced approach [38, 39]. Age and gender are the most common obligate variables because they are easy to acquire in the real world and adding them usually does not increase the clinical burden [40–42]. Hence, we extracted “age” and "gender" as the factors in our predictive model of III to IV aGVHD.

We observed that our predict model was associated with grade III to IV and grade II to IV gut aGVHD after HID HSCT, which suggested that routine GVHD prophylaxis methods were not sufficient to prevent severe gut aGVHD in high-risk patients. Severe gut aGVHD is difficult to treat and is the greatest cause of GVHD-related mortality [43]. Thus, our predicted model could help to direct more intense prophylaxis for gut aGVHD in high-risk patients after HID HSCT with ATG for GVHD prophylaxis.

The present study had some limitations. First, the model was not associated with the development of grade III to IV liver aGVHD after HID HSCT, which might be due to the small sample of severe liver aGVHD in the present study. However, we observed that the rate of grade I to IV liver aGVHD in high-risk group was higher than that of low-risk group. Second, although we verified the model successfully in the validation cohort, this was a single-center study and the sample of validation cohort was relatively small. Third, ATG was administered to prevent GVHD in this research, but ATG is contained in 94 per cent of conditioning regimens for HID HSCT in China. Thus, the predicted value of our model should be further confirmed in patients receiving HID HSCT with PTCY for GVHD prophylaxis and in those receiving identical sibling or unrelated donor HSCT. Thus, the model should be further evaluated by independent cohorts in multicenter studies. Lastly, we did not monitor plasma cytokines (e.g., interleukin [IL]-2) and biomarkers (e.g., ST2, REG3α, TNFR1, and IL-2Rα) [44, 45], which may further improve the efficacy of our predicted model.

## Conclusions

We established a comprehensive model which could predict the development of severe aGVHD in HID HSCT recipients. This was the first predicted model for severe aGVHD which can be popularized easily, can help to provide risk-stratification directed aGVHD prophylaxis, and may further decrease the risk of severe aGVHD in HID HSCT recipients. In future, prospective, multicenter studies can further confirm the efficacy of our predicted model.

## Supplementary Information

**Additional file 1. **Additional methods, Additional tables S1–S3 and additional figures S1–S4.

## Data Availability

The datasets generated during the analysis of the current study are available from the corresponding author on reasonable request.
