# Supplementary material for: A comprehensive model to predict severe acute graft-versus-host disease in acute leukemia patients after haploidentical hematopoietic stem cell transplantation
Source: Exp Hematol Oncol. 2022 May 3;11:25. doi: 10.1186/s40164-022-00278-x (PMC9067003; doi:10.1186/s40164-022-00278-x)
Supplement: Supplementary file 1 — Additional file 1. Additional methods, Additional tables S1–S3 and additional figures S1–S4. [file 40164_2022_278_MOESM1_ESM.docx]

**Addiitonal file 1: Appendix S1**

**Title: A comprehensive model to predict severe acute graft-versus-host disease in acute leukemia patients after haploidentical hematopoietic stem cell transplantation**

**Journal name: Experimental Hematology & Oncology**

**Authors:** Meng-Zhu Shen^1*^, Shen-Da Hong^2*^, Rui Lou^1*^, Rui-Ze Chen^1,3*^, Xiao-Hui Zhang^1^, Lan-Ping Xu^1^, Yu Wang^1^, Chen-Hua Yan^1^, Huan Chen^1^, Yu-Hong Chen^1^, Wei Han^1^, Feng-Rong Wang^1^, Jing-Zhi Wang^1^, Kai-Yan Liu^1^, Xiao-Jun Huang^1,4,5^, and Xiao-Dong Mo^1,5^.

**Affiliations:** ^1^Peking University People's Hospital, Peking University Institute of Hematology, National Clinical Research Center for Hematologic Disease, Beijing Key Laboratory of Hematopoietic Stem Cell Transplantation, Beijing, China;

^2^ National Institute of Health Data Science at Peking University, Peking University Health Science Center, Beijing, 100191, China.

^3^ Department of Hematology, Jiangsu Province Hospital, The First Affiliated Hospital of Nanjing Medical University;

^4^ Peking-Tsinghua Center for Life Sciences, Academy for Advanced Interdisciplinary Studies, Peking University, Beijing, China;

^5^ Research Unit of Key Technique for Diagnosis and Treatments of Hematologic Malignancies, Chinese Academy of Medical Sciences, Beijing 2019RU029, China;

^*^Meng-Zhu Shen, Shen-Da Hong, Rui Lou, Rui-Ze Chen contributed equally to this manuscript.

**Correspondence:** Xiao-Dong Mo; Peking University People’s Hospital, Peking University Institute of Hematology, No. 11 Xizhimen South Street, Xicheng District, Beijing 100044, China; Tel: 8610-8832-6001; Fax: 8610-8832-4577; E-mail: mxd453@163.com

**Contents**

**Additional methods**

Protocol for GVHD prophylaxis ………………………………………………………………………...3

Evaluation of graft composition………………………………………………………………………….4

Definitions……………………………………………………………………………………………......5

Variables for building machine learning models……………………………………………………....…6

**Additional Tables**

Table S1. Generalized Linear Model Regression Results…………………………………………..….....7

Table S2. Table of confusion in the training cohort…………………………………………………........8

Table S3. Table of confusion in the validation cohort………………………………………………….…9

**Additional Figures**

Figure S1. p-value iteration during backward feature selection...…………………………………….....10

Figure S2. The 100-day cumulative incidence of grade III to IV aGVHD in the low- and high-risk groups in patients with HCT-CI scores of 0..…………………………………………………………………....11

Figure S3. The 100-day cumulative incidence of grade III to IV aGVHD in the low- and high-risk groups in patients with HCT-CI scores ≥1..………………………………………………………………….....12

Figure S4. The clinical outcomes at 100 days after HSCT in the low-risk and high-risk group.………....13

**References**

References………………………………………………………………………………………………14

**Protocol for graft-versus-host disease (GVHD) prophylaxis**

All the HID HSCT recipients received rabbit antithymocyte globulin (ATG, thymoglobulin, 2.5 mg/kg/day, days −5 to −2; Sanofi, France), cyclosporine A (CsA), mycophenolate mofetil (MMF), and short-term methotrexate (MTX) for GVHD prophylaxis (day 0 being the first day of donor cell infusion). CsA (2.5 mg/kg, q12h, intravenous [i.v.]) was used from day −3, of which the trough concentration was adjusted to 150–250 ng/mL. It was switched to oral administration when the patient’s bowel function returned to normal. From day −3, 0.25–0.5 g of MMF was administered orally every 12 h, then it was discontinued when neutrophil engraftment was achieved. Following graft infusion, a dose of 15 mg/m^2^ of MTX was administered i.v. on day +1, as well as a dose of 10 mg/m^2^ on days +3, +5, and +11. Particularly, patients with maternal donors or collateral relative donors could receive two doses of 14.5 mg/kg cyclophosphamide on days +3 and +4 post-HSCT based on ATG (n=44) [1].

**Evaluation of graft composition**

Samples from grafts were stained with MoAb for flow cytometry analysis of the following surface Ags: CD45, CD3, CD4, CD8, CD14, CD34. They were performed at Peking University People’s Hospital, essentially the same as previous reports [2, 3]. Acquisition and analyses were performed with FACS Diva 6.0 (Becton-Dickinson, San Jose, CA, USA).

**Definitions**

Disease risk index (DRI) was defined and graded according to the criteria of Armand et al. [4, 5]. The neutrophil engraftment was defined as the first of 3 consecutive days that the absolute neutrophils achieved 0.5×10^9^/L without G-CSF, and platelet engraftment was defined as the first of 7 consecutive days that the absolute platelets achieved 20×10^9^/L with transfusion independence. Relapse was defined as morphologic evidence of disease in peripheral blood, bone marrow, or extramedullary samples. Leukemia-free survival (LFS) was defined as the survival period with continuous complete remission (CR) after transplantation. Non-relapse mortality (NRM) was defined as death without relapse after transplantation. Overall survival (OS) was the period between the date of transplantation and death from any cause. GVHD was diagnosed and graded according to internationally accepted criteria [6, 7].

**Variables for building machine learning models**

Variables included age, age group, gender, disease status before HSCT, disease risk index (DRI) before HSCT, hematopoietic cell transplantation-specific comorbidity index (HCT-CI) before HSCT, donor age, donor/recipient relation, donor/recipient gender matched, cytomegalovirus (CMV) serostatus, human leukocyte antigen (HLA) disparity, blood group compatibility, conditioning regimen, engraftment, mononuclear cells (MNC), CD34+ cell counts, CD3+ cell counts, CD4+ cell counts, CD8+ cell counts, CD14+ cell counts, CD8+/CD3+ cells ratio, CD4+/CD8+ cells ratio, CD4+/CD3+ cells ratio, and CD3+/CD14+ cells ratio in the grafts.

**Variables for training machine learning models**

| Variables |  |
| --- | --- |
| Age (years) | actual numerical value |
| Age group | <18=0; ≥18=1 |
| Gender | male=0; female=1 |
| Disease status before HSCT | CR1=0; > CR1=1 |
| DRI | low and intermediate risk=0; high and very high risk=1 |
| HCT-CI score | actual numerical value |
| Donor age (years) | actual numerical value |
| Donor/recipient gender matched | others=0; female donor/male recipient=1 |
| Donor/recipient relation | immediate relative donors, others=0; immediate relative donors, maternal donors=1; collateral relative donors=2 |
| Blood group compatibility | mismatched=0; matched=1 |
| CMV serostatus | D+/R+=0; D+/R-=1; D-/R+=2; D-/R-=3 |
| HLA disparity | 1 locu=0; ≥2 loci=1 |
| Conditioning regimen | TBI-based=0; Chemotherapy-based=1 |
| Engraftment | No=0; Yes=1 |
| Mononuclear cell counts (×10^8^/kg) | actual numerical value |
| CD34^+^ cell counts (×10^6^/kg) | actual numerical value |
| CD3^+^ cell counts (×10^6^/kg) | actual numerical value |
| CD4^+^ cell counts (×10^6^/kg) | actual numerical value |
| CD8^+^ cell counts (×10^6^/kg) | actual numerical value |
| CD14^+^ cell counts (×10^6^/kg) | actual numerical value |
| CD8^+^/CD3^+^ cells ratio | actual numerical value |
| CD4^+^/CD8^+^ cells ratio | actual numerical value |
| CD4^+^/CD3^+^ cells ratio | actual numerical value |
| CD3^+^/CD14^+^ cells ratio | actual numerical value |

CMV, cytomegalovirus; CR, complete remission; D, donor; DRI, disease risk index; HCT-CI, hematopoietic cell transplantation-specific comorbidity index; HLA, human leukocyte antigen; HSCT, hematopoietic stem cell transplant; R, recipient; TBI, total body irradiation.

**Table S1. Generalized Linear Model Regression Results**

| **Variables** | **Coef** | **Std err** | **Z** | ***P***  **value** | **95%CI** | |
| --- | --- | --- | --- | --- | --- | --- |
|  |  |  |  |  | **0.025** | **0.975** |
| Const | -2.9046 | 0.900 | -3.228 | 0.001 | -4.668 | -1.141 |
| Donor/recipient relation | -0.5829 | 0.412 | 1.416 | 0.157 | -0.224 | 1.390 |
| CD8+ cell counts (×10^6^/kg) | -0.0089 | 0.006 | -1.596 | 0.110 | -0.020 | 0.002 |
| CD3+/CD14+ cells ratio | 0.8371 | 0.377 | 2.219 | 0.026 | 0.098 | 1.576 |
| Gender | 0.7965 | 0.491 | 1.624 | 0.104 | -0.165 | 1.7589 |
| Age (years) | -0.0288 | 0.017 | -1.733 | 0.083 | -0.061 | 0.004 |

CI, confidence interval; const, constant.

Donor/recipient relation: immediate relative donors, others: 0; immediate relative donors, maternal donors: 1; collateral relative donors: 2;

Gender: male: 0; female: 1;

Age, CD8+ cell counts, CD3+/CD14+ cells ratio: actual numerical value

**Table S2. Table of confusion in the training cohort**

|  | Predicted  grade III-IV GVHD negative | Predicted  grade III-IV GVHD positive |
| --- | --- | --- |
| Grade III-IV GVHD  negative | 215  (True negative) | 101  (False positive) |
| Grade III-IV GVHD  positive | 7  (False negative) | 12  (True positive) |

**Table S3. Table of confusion in the validation cohort**

|  | Predicted  grade III-IV GVHD negative | Predicted  grade III-IV GVHD positive |
| --- | --- | --- |
| Grade III-IV GVHD  negative | 92  (True negative) | 29  (False positive) |
| Grade III-IV GVHD  positive | 7  (False negative) | 7  (True positive) |

**Fig. S1. p-value iteration during backward feature selection**


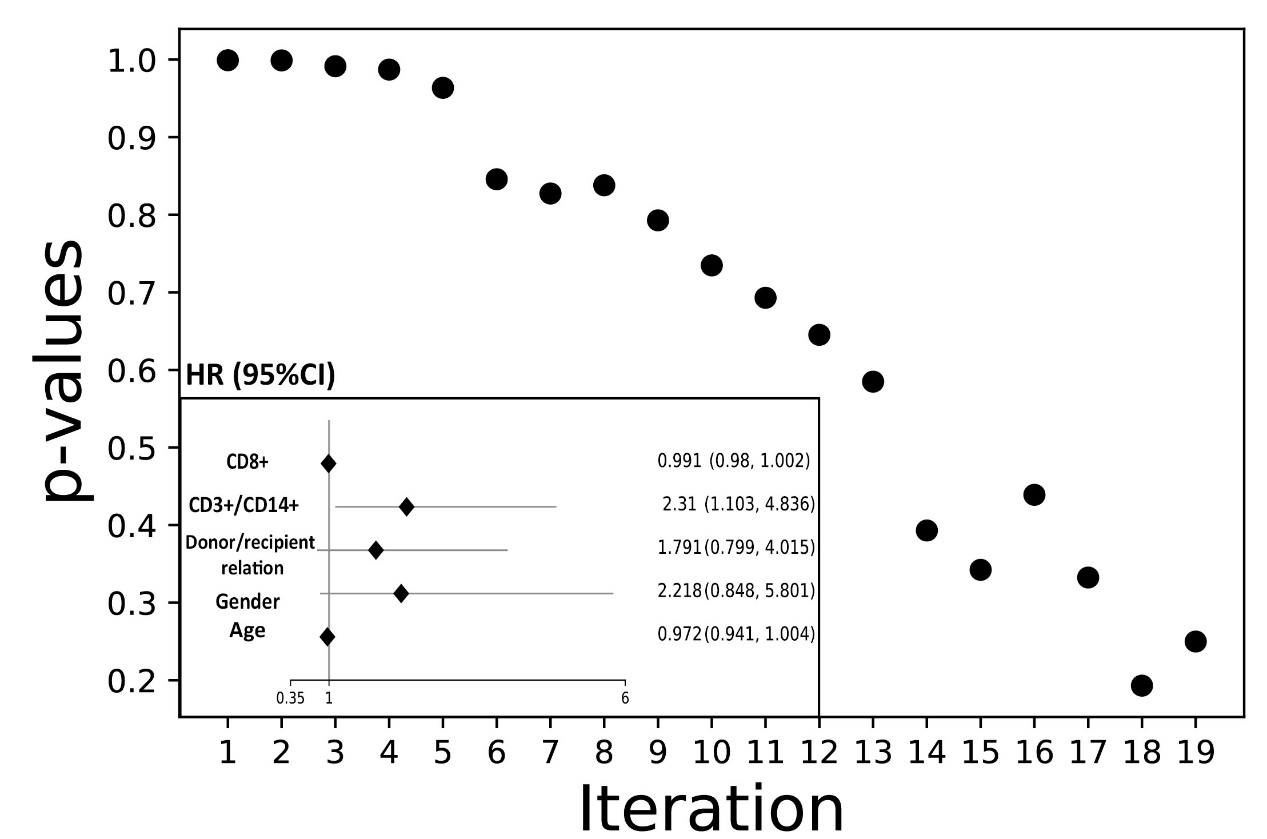


**Fig. S2. The 100-day cumulative incidence of grade III to IV aGVHD in the low- and high-risk groups in patients with HCT-CI scores of 0**


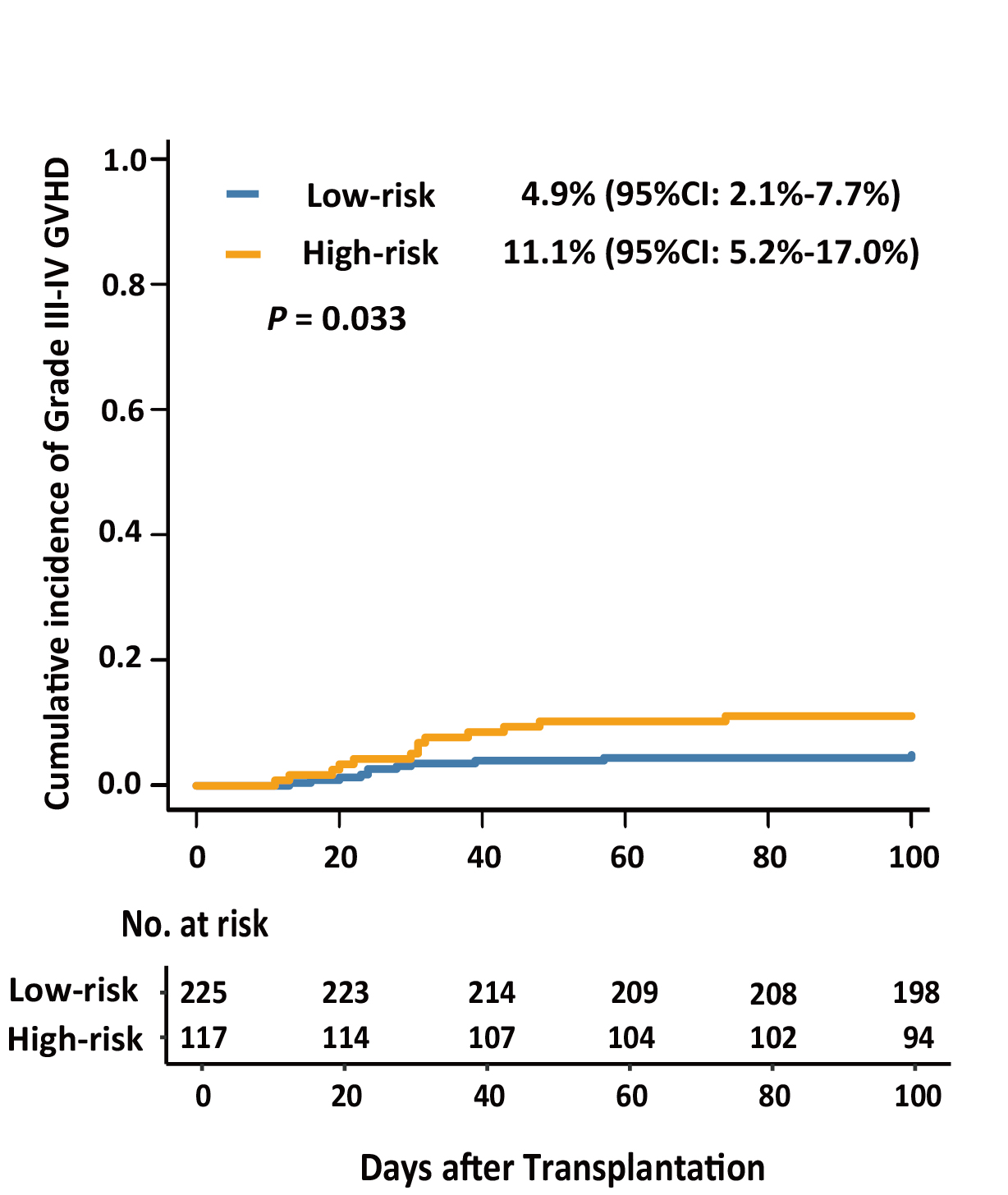


**Fig. S3. The 100-day cumulative incidence of grade III to IV aGVHD in the low- and high-risk groups in patients with HCT-CI scores ≥ 1**


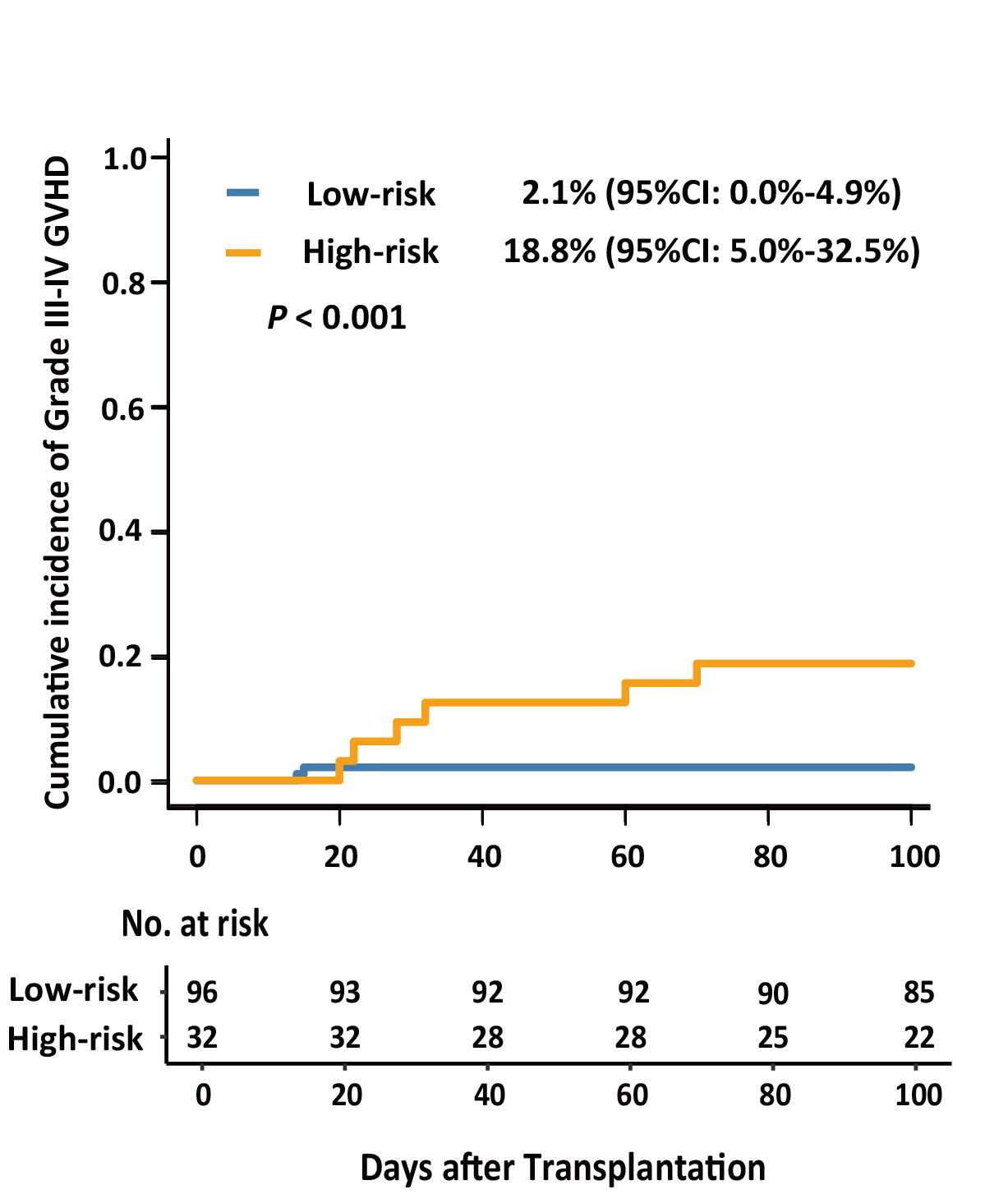


**Fig. S4. The clinical outcomes at 100 days after HSCT in the low-risk and high-risk group.** The 100-day cumulative incidence of relapse (A), non-relapse mortality (B), leukemia-free survival (C) and overall survival (D).


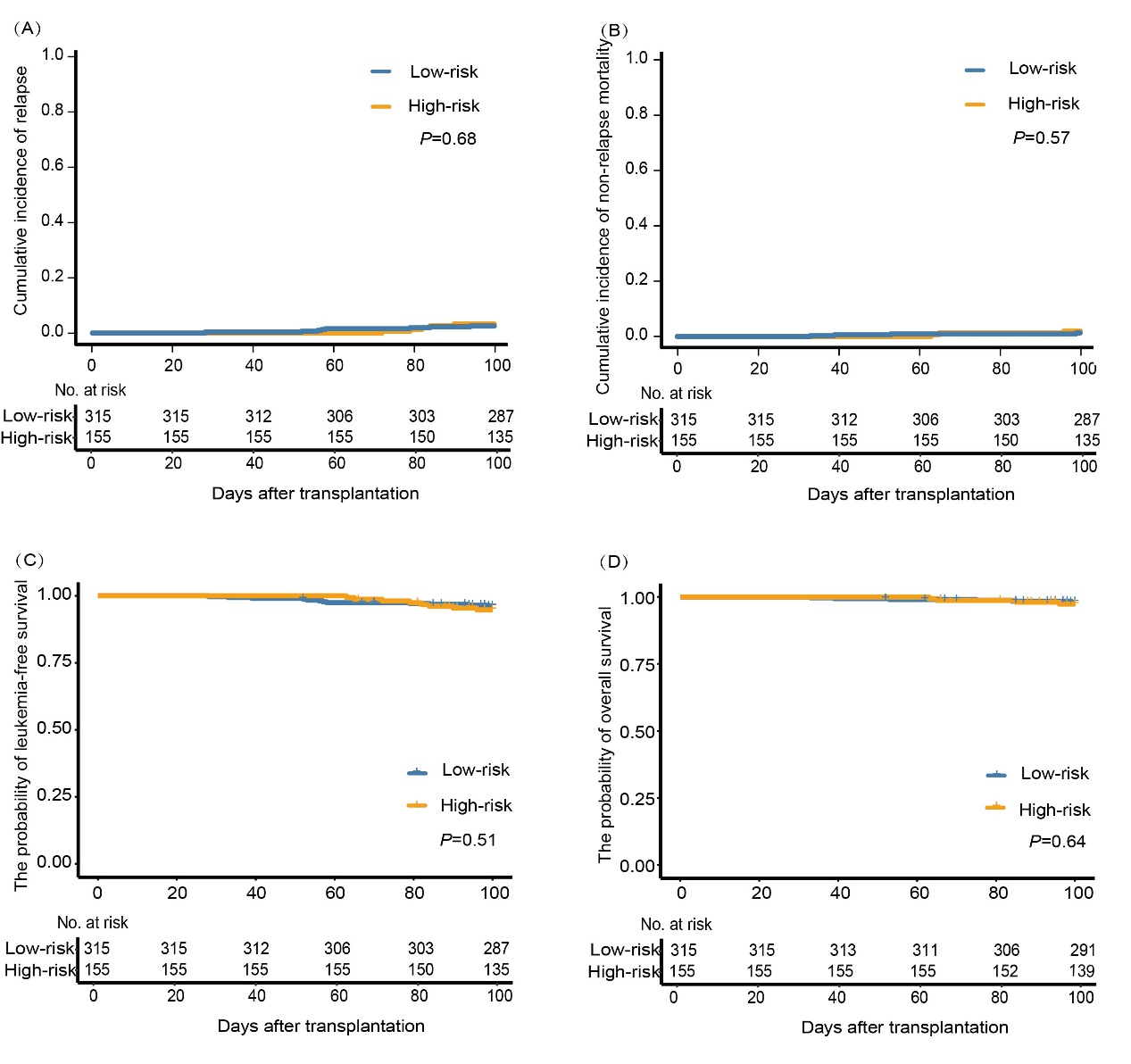


**References:**

1. Wang Y, Wu DP, Liu QF, Xu LP, Liu KY, Zhang XH, et al. Low-dose post-transplant cyclophosphamide and anti-thymocyte globulin as an effective strategy for GVHD prevention in haploidentical patients. J Hematol Oncol. 2019;12(1):88.

2. Liu Y, Chen S, Yu H. Standardization and Quality Control in Flow Cytometric Enumeration of CD34(+) cells. Zhongguo Shi Yan Xue Ye Xue Za Zhi. 2000;8(4):302-6.

3. Luo XH, Chang YJ, Xu LP, Liu DH, Liu KY, Huang XJ. The impact of graft composition on clinical outcomes in unmanipulated HLA-mismatched/haploidentical hematopoietic SCT. Bone Marrow Transplant. 2009;43(1):29-36.

4. Armand P, Kim HT, Logan BR, Wang Z, Alyea EP, Kalaycio ME, et al. Validation and refinement of the Disease Risk Index for allogeneic stem cell transplantation. Blood. 2014;123(23):3664-71.

5. Mo XD, Zhang XH, Xu LP, Wang Y, Yan CH, Chen H, et al. Disease Risk Comorbidity Index for Patients Receiving Haploidentical Allogeneic Hematopoietic Transplantation. Engineering. 2021;7(2):162-9.

6. Przepiorka D, Weisdorf D, Martin P, Klingemann HG, Beatty P, Hows J, et al. 1994 Consensus Conference on Acute GVHD Grading. Bone Marrow Transplant. 1995;15(6):825-8.

7. Jagasia MH, Greinix HT, Arora M, Williams KM, Wolff D, Cowen EW, et al. National Institutes of Health Consensus Development Project on Criteria for Clinical Trials in Chronic Graft-versus-Host Disease: I. The 2014 Diagnosis and Staging Working Group report. Biol Blood Marrow Transplant. 2015;21(3):389-401.e1.
